# Circulating tumour cell combined with DNA methylation for early detection of hepatocellular carcinoma

**DOI:** 10.3389/fgene.2022.1065693

**Published:** 2022-11-21

**Authors:** Wenjin Liang, Zhigao Xu, Fangyu Kong, Xiao Huang, Yuxin Xiao, Wei Zhou, Shaojun Ye, Qifa Ye

**Affiliations:** Hubei Key Laboratory of Medical Technology on Transplantation, Zhongnan Hospital of Wuhan University, Institute of Hepatobiliary Diseases of Wuhan University, Transplant Center of Wuhan University, Wuhan, Hubei, China

**Keywords:** circulating tumor cell, cell free DNA, DNA methylation, GNB4, Riplet, hepatocellular carcinoma, early diagnosis

## Abstract

**Background:** The inadequate early detection strategies makes hepatocellular carcinoma (HCC) patients with poor prognisis. Therefore, more effective detection methods are urgently needed for early detection and early intervention of HCC.

**Methods:** 17 cases of suspected HCC patients and 11 cases of HBV-related decompensated cirrhosis (HBV-DeCi) patients were enrolled. For each patient, 5 ml blood sample was separated into circulating tumor cells (CTCs) and plasma, CTCs were stained with Diff staining for counting. Plasma was used for extracting cell free DNA (cfDNA) and then analyzed by qMSP assay. Ct values were recorded for GNB4 and Riplet as target genes and β-actin as an endogenous reference gene. Finally, clinical efficacy of CTC count combined with GNB4/Riplet methylation detection for early diagnosis of HCC was analyzed.

**Results:** The CTC of HCC patients has pleomorphic characteristics, but it is difficult to distinguish from other blood cells with non-obviously pleomorphic of CTC. Although a small number of CTCs can also be detected in HBV-DeCi patients (control group), the number is significantly lower than that in HCC patients, the sensitivity and specificity of CTC for HCC detection were 70.6% and 90.9% (AUC = 0.81). The Ct values of GNB4 and Riplet methylation were significantly different between HCC patients and control group patients. When CTC combined with two genes, the AUC value was significantly increased to 0.98, the sensitivity was 88.2%, and the specificity was 100%.

**Conclusion:** Our study has developed a novel test that CTC count combined with GNB4/Riplet methylation detection and showed its high performance for early diagnosis of HCC.

## Introduction

Hepatocellular carcinoma (HCC) is a leading cause of cancer-related death worldwide, in part due inadequate early detection strategies (Bruix et al., 2019). Current recommendations for screening consist of semiannual abdominal ultrasound with or without serum alpha fetoprotein (AFP) in patients with cirrhosis and in demographic subgroups with chronic hepatitis B infection. However, this screening strategy has several deficiencies, including suboptimal early-stage sensitivity, false positives with subsequent harms, inter-operator variability in ultrasound performance, and poor adherence (Singal et al., 2022). A meta-analysis of cohort studies reported the sensitivity of ultrasound for early-stage HCC detection is only 45%, which increases to 63% with the addition of AFP (Tzartzeva et al., 2022). Therefore, more effective detection methods are urgently needed for early detection and early intervention of HCC progression.

Currently, it has been found that the liquid biopsies, with sufficient performance characteristics and blood-based biomarkers for early-stage disease, could overcome several of these barriers to improving early-stage detection (Johnson et al., 2022). Also, it have emerged as a minimally invasive technique to improve early cancer detection (Heitzer et al., 2019). For HCC, identifying tumor-specific biomarkers, including Circulating tumor cell (CTC), cell-free DNA (cfDNA), methylated DNA, through liquid biopsies have show the promise for early diagnosis and therapeutic outcome (Parikh et al., 2022). It has been shown that, with a novel integrated immunomagnetic microfluidic platform (iMAC), the distinct stem-related markers’ expression of circulating tumor initiating cells (CTICs) could distinguish primary HCC, recurrent HCC, and TACE-resistant HCC (Zheng et al., 2022). CfDNA in peripheral blood contains circulating tumor DNA (ctDNA) that reflects molecular abnormalities in tumor tissue. The potential of cfDNA/ctDNA can be investigated as biomarkers for predicting the therapeutic outcome in unresectable hepatocellular carcinoma (u-HCC) patients treated with anti-PD-L1/VEGF therapy (Matsumae et al., 2022).

The cfDNA in blood is a DNA fragment of about 150-200 bp in length, which is derived from apoptotic or necrotic cells, including tumor cells (Li et al., 2021). Aberrant DNA methylation may be one of the early events in tumor formation, which can reflect early changes in tumor (Luo et al., 2022). Therefore, analysis of cfDNA methylation profiles in blood can be used for early diagnosis of cancer, postoperative monitoring, and prediction of patient prognosis (Lo et al., 2021). One study have constructed a HCC diagnostic model consisting of six plasma DNA methylation markers, with a sensitivity of 91% and a specificity of 92% for the diagnosis of BCLC stage 0/A HCC (Kisiel et al., 2019). Therefore, liquid biopsy targeting CTC and cfDNA has attracted more and more attention in the clinical application of tumor diagnosis. However, currently, research on CTC combined with cfDNA in the early diagnosis of HCC has not been found.

In our study, Through the analysis of LIHC methylation 450 K chip in The Cancer Genome Atlas (TCGA) database and Gene Expression Omnibus (GEO), we have found that the combination of G protein subunit beta 4 (GNB4) and Riplet methylation can act as a role for the diagnostic performance of HCC (unpublished data). Furthermore, through the CTC counting combined with GNB4/Riplet methylation detecting, we detect suspected HCC patients, and then analyze the correlation between the detection results and clinical characteristics. So as to explore the clinical efficacy of CTC count combined with GNB4/Riplet methylation detection in improving the early diagnosis of HCC.

## Materials and methods

### Patients and samples

This study was approved by the Ethics Committee of Zhongnan Hospital of Wuhan University (NO.2020108) and all participants signed informed consent formulated under the Declaration of Helsinki principles. 17 cases of suspected HCC patients, who underwent hepatectomy or liver transplantation were enrolled in Zhongnan Hospital of Wuhan University from May 2020 to May 2022, with the age between 27 and 85 years, according to the postoperative pathological diagnosis, 17 HCC patients were divided into three groups: well, moderate and poor differentiation. Meanwhile, 11 cases of patients with HBV-DeCi were enrolled in the control group. The plasma samples were collected before surgery at the same time as CTC sampling. The clinical and pathological data included sex, age, tumor diameter, tumor differentiation, AFP, and HBsAg.

### CTCs isolation and diff staining

The 5 ml blood sample of patient was diluted up to 15 ml with physiological saline containing 8% PFA 375 μl, and then stood for 10min. CTCs were isolated by CTCBIOPSY^®^ device (Wuhan YZY Medical Science and Technology Co., Ltd., Wuhan, China), which was composed of a membrane with 8 μm size pores. The captured cells on the membrane were stained with Diff staining.

There were six criteria of CTCs characteristics for evaluating cells captured in peripheral blood: 1) the nuclear atypia: irregularity of nuclear shape, may be nodular or lobulated *etc.*; 2) the nuclear-cytoplasmic ratio was higher than 0.8; 3) a large cell diameter (the long diameter): >15 μm; 4) the hyperchromatic nucleus and nonhomogeneous staining (due to the increase of chromatin and the thicker particles in cancer cells, the nucleus was hyperchromatic); 5) the nuclear membrane was thickened, sunken and wrinkled; 6) a large nucleoli or abnormal nuclear division. Abnormal cells captured by this method were identified as CTCs only if they met no less than four criteria above, or met the sixth criterion and any other two criteria. If they met any three criteria except the sixth criterion, or met only the sixth criterion, they were identified as the suspected CTCs. All candidates CTCs were blindly reviewed and identified independently by two senior cytopathologists.

### Bioinformatics analysis

The TCGA-LIHC methylation 450 k chip (Normal = 50, Tumor = 377) was performed differential methylation analysis to obtain 1,148 probes (rank-sum test, *p* < 0.05; β-Tumor/β-Normal≥2; β-Tumor≥0.3, β-Normal≤0.1). Multiple 450 K chips (GSE136319, GSE89852, GSE83691, and GSE77269) related to HCC in the GEO database were combined into a single GEO dataset for validation of the above 1,148 probes, of which 447 were significantly different in the GEO dataset (rank-sum test, *p* < 0.05). To exclude the interference of blood cells, we further evaluated the methylation levels of these 447 probes on whole blood samples from healthy people (GSE40279, *n* = 656), and selected the top 30 probes with the smallest β values. The HCC-specific probes were further selected, the average methylation β values of the top 30 probes on other cancer types in TCGA were calculated, and 0.2 was used as the threshold. Finally, GNB4 and Riplet were selected for this study. The screening process is shown in Figure 2A, the methylation of GNB4 and Riplet in TCGA-LIHC is shown in Figure 2B.

### DNA extraction and bisulfite conversion

The plasma collected after CTC separation from whole blood was centrifuged again for 10 min (2,000rcf), then transferred to a new 5 ml centrifuge tube and frozen at −80°C, cfDNA was extracted with nucleic acid extraction reagent (AA16, Wuhan Ammunition Life-tech Company, Ltd.). For details, please refer to the kit instructions. The plasma volume was 2 ml and the elution volume was 50 μl. The extracted DNA was bisulfite converted with nucleic acid purification reagent (AA20, Wuhan Ammunition Life-tech Company, Ltd.). The amount of cfDNA used for transformation was 40 μl, and the final elution volume was 50 μl.

### methylation-specific polymerase chain reaction

According to the instructions of the Plasma DNA Methylation Detection Kit for Liver Cancer (Real-Time PCR) (Wuhan Ammunition Life-tech Company, Ltd.) and information about the vendor at http://www.ammulifetech.com/, methylation-specific PCR was used to detect the methylation levels of GNB4 and Riplet in the samples. Amplification reactions were performed on an ABI 7500 fluorescence PCR instrument. The 50 μl mixed reaction solution contains: 6 μl PCR reaction solution I (PCR buffer solution), 3.4 μl PCR reaction solution II (primers and probes for GNB4, Riplet and β-actin), 0.6 μl Taq enzyme and 40 μl bisulfite converted DNA. The reaction program was as follows: 5min at 95°C followed by 45 cycles of 15 s at 95°C and 30 s at 60°C.

If the Ct value of the internal control gene β-actin of the sample is ≤35, the sample is valid, otherwise the sample is determined to be invalid. If the Ct value of the target gene GNB4 and Riplet is ≤43, and the amplification curve shows a S-shaped trend, the methylation of GNB4 and Riplet is positive. If the Ct value is >43 or cannot be detected, the methylation of GNB4 and Riplet is negative. If the methylation of GNB4 or Riplet is positive, the result of the sample is judged to be positive. If the methylation of both GNB4 and Riplet is negative, the result is judged to be negative.

### Statistical analysis

The logistic regression algorithm was applied to model construction and the Ct value was used for ROC analysis, and the maximum value of the Youden index was taken as the best cut-off value to estimate the area under the ROC curve (AUC) value. Respectively, the sensitivity and specificity to distinguish HCC samples from control group. Statistical analyses were performed using GraphPad Prism 8 (GraphPad Software, Inc., San Diego, CA, United States) and SPSS v.18.0 (SPSS Inc., Chicago, IL, United States). All bioinformatics were performed using R version 3.6.1. Kruskal–Wallis Test was used for data in multiple groups. All data are presented as mean ± SD. Pairwise comparisons were performed based on the two-tailed Student’s t-test. A *p*-value of <0.05 was considered statistically significant.

## Results

### CTC diagnostic test has good clinical application efficacy for HCC patients

In order to confirm the diagnosis of HCC in early clinical stage, we collected 5 ml whole blood from patients with suspected HCC during admission and before surgery, and then performed CTC detection. A total of 17 patients with HCC were enrolled in this study. The details of clinical and tumor characteristics for the 17 patients as was shown in Table 1. The mean patient age was 54 ± 11 years (range, 27–85 years). Of the patients, 94% (16/17) were male and 6% (1/17) were female. The tumor diameter was 6.3 ± 4.76 cm (range, 1.2–17 cm). The AFP was 3,158.6 ± 7,200.9 ng/ml (range, 1.9–11053.3 ng/ml). The CTC count was 5 ± 4.1 (range, 0–16). In all, the well, moderate and poor differentiation of HCC are respectively as 24, 47, and 29%. Hepatitis B surface antigen (HBsAg) was positive in 88% (15/17) of the patients.

**TABLE 1:** Clinical Characteristics of 17 hepatocellular carcinoma patients.

Clinical characteristics	No. of patients
Age (years)	Mean 54 ± 11 (27–85)
	Median 55
Sex	
Male	16
Female	1
Tumor diameter (cm)	Mean 6.3 ± 4.76 (1.2–17)
	Median 5.5
AFP (ng/ml)	Mean 3,158.6 ± 7,200.9 (1.9–11,053.3)
	Median 276.1
CTC count	Mean 5 ± 4.1 (0–16)
	Median 5
Tumor differentiation	
Well	4
Moderate	8
Poor	5
HBsAg	
Negative	2
Positive	15

**FIGURE 1:**
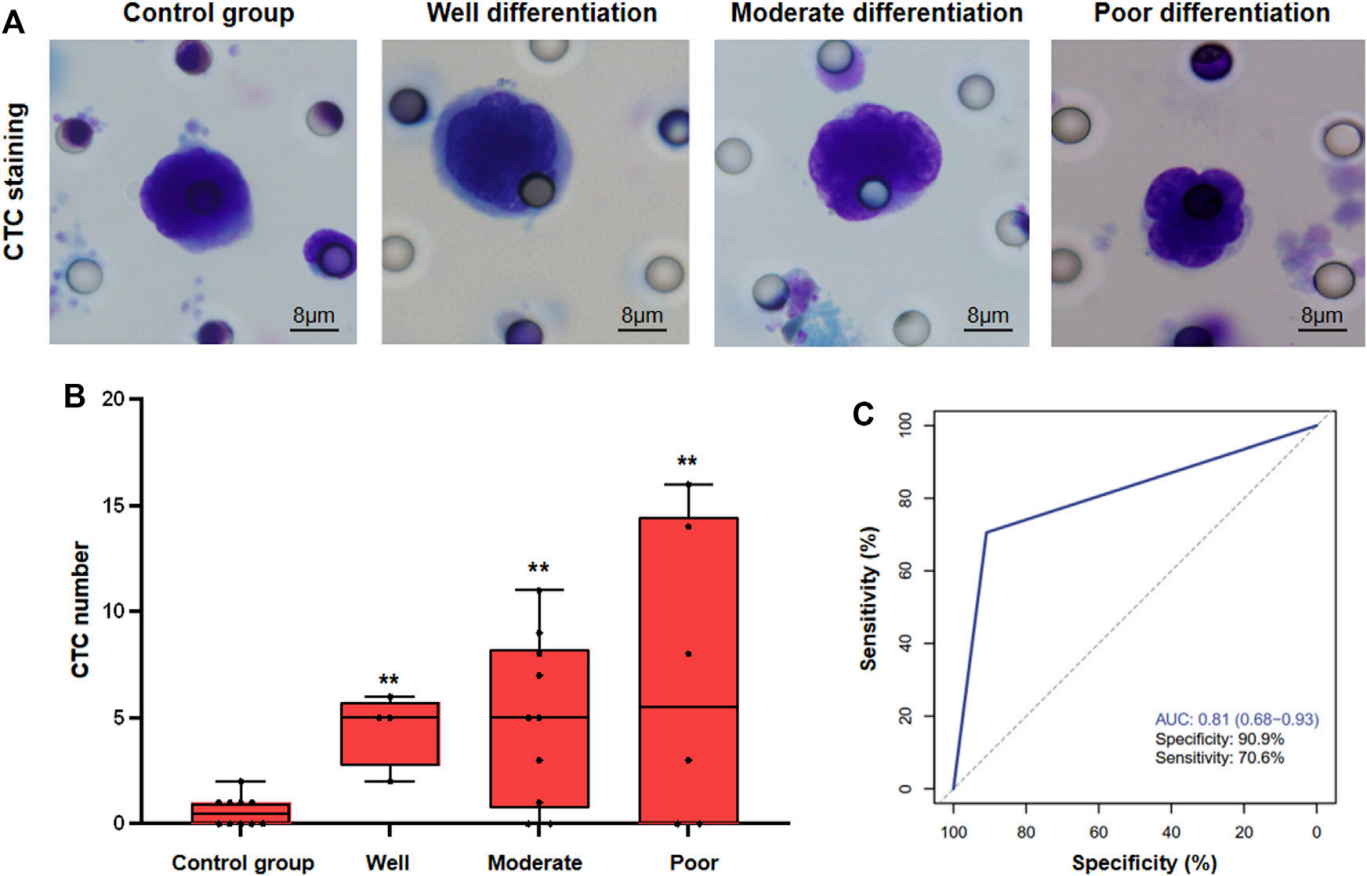
CTC detection in HCC patients. **(A)**, The typical microscopic images of CTC in different groups of patients **(B)**, Statistical analysis of CTC count in different groups of patients. **(C)**, ROC analysis of CTC detection (** vs. Control group, *p* < 0.01).

Furthermore, In order to verify the detection efficiency of CTC, 11 HBV-DeCi patients were included as the control group, and the number of CTCs in the 11 control group was also detected. Besides, we have found the following pathological features of CTCs: 1, single scattered hepatocellular carcinoma CTCs have a larger diameter than 15 microns; 2, the proportion of nucleoplasm increased, and there was obvious cell atypia; 3, the nucleus has folds, some have nuclear furrow, some nucleoli are obvious; 4, The cytoplasm is bitropic. The histopathological characteristics of CTCs in HCC are relatively simple, and the pleomorphism is not obvious. The typical microscopic images of CTC were shown in Figure 1A. Meanwhile, we have found that although a small number of CTCs can also be detected in HBV-DeCi patients, the number is significantly lower than that in HCC patients. For HCC patients with different degrees of differentiation, there was no significant difference in CTC detection count (Figure 1B). Furthermore, we have found that the sensitivity and specificity of CTC for HCC detection were 70.6% and 90.9% (AUC = 0.81). It shows that, for HCC patients, CTC diagnostic test can have the good clinical application efficacy.

### The plasma methylation levels of GNB4 and Riplet in HCC patients were significantly higher than those in HBV-DeCi patients

**FIGURE 2:**
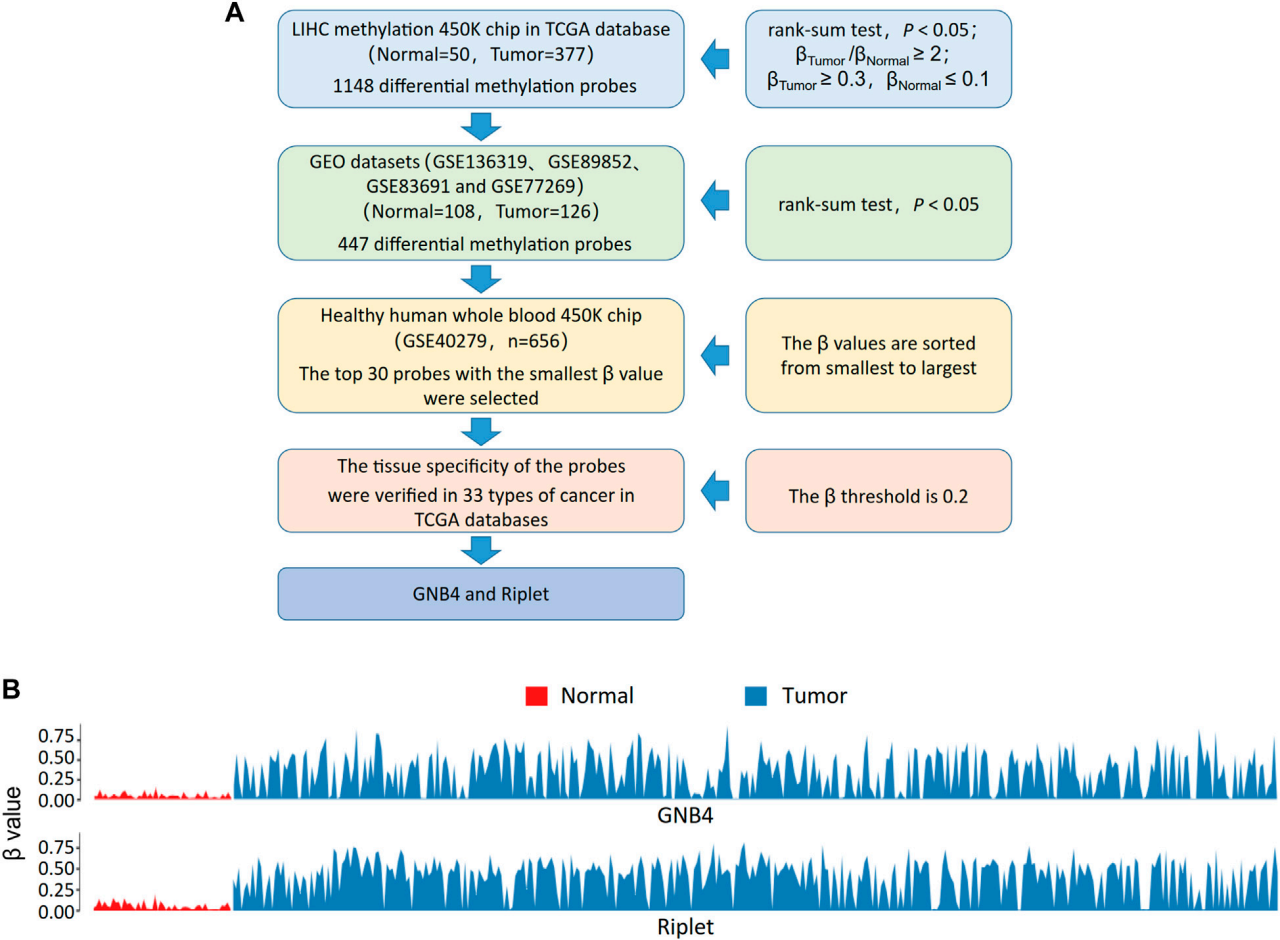
Methylation of GNB4/Riplet is selected by bioinformatics analysis. **(A)**, The screening process for picking out GNB4 and Riplet. **(B)**, the methylation of GNB4 and Riplet in TCGA-LIHC.

In order to further increase the diagnostic efficiency of blood testing in HCC patients, we continued to collect plasma from HCC patients after CTCs were sorted. The specific methylation gene probes in TCGA-LIHC were selected by bioinformatics method, GNB4 and Riplet were finally selected for this study (Figures 2A,B).

**FIGURE 3:**
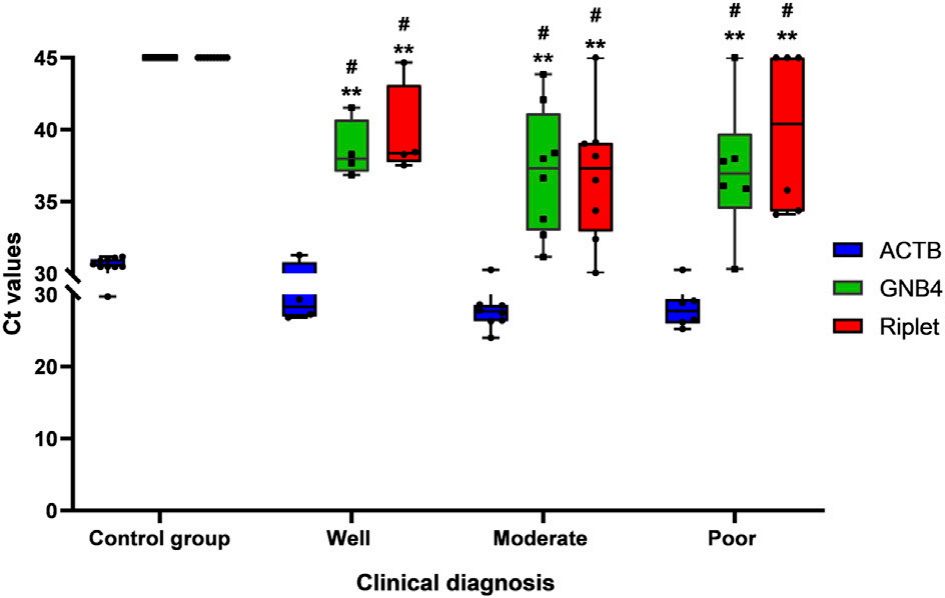
Distribution of Ct value of GNB4 and Riplet in well, moderate and poor differentiation of HCC (^**#**^ vs. Control group, *p* < 0.05; ** vs. ACTB, *p* < 0.01).

In further operation, we used MSP to detect GNB4 and Riplet gene methylation levels in plasma samples from 17 HCC patients and 11 control group patients, and then analyzed the distribution of Ct values of GNB4 and Riplet in samples of HCC patients with different degrees of differentiation. As is shown in Figure 3. The Ct values of the two genes were significantly different between HCC and control group patients, but for HCC patients, there was no significant difference in the Ct values of GNB4 and Riplet genes in the well, moderate and poor differentiation samples. The above result indicates that GNB4 and Riplet gene methylation levels were significantly increased in HCC patients, however, the methylation level was not affected by the differentiation degree of HCC.

### The combined detection of GNB4 and Riplet gene methylation levels can distinguish HCC from HBV-DeCi patients plasma

To further understand the clinical diagnostic efficacy of combined detection of GNB4 and Riplet gene methylation levels in HCC patients, we performed a combined analysis of the two genes. Table 2 shows the statistical characteristics of Ct values of GNB4, Riplet and endogenous gene β-actin. The mean Ct values of GNB4 gene in HCC patients and HBV-DeCi patients were 37.44 and 44.46, and the mean Ct values of Riplet gene were 38.49 and 45 respectively. These results indicated that Ct values of the two genes were significantly different between HCC patients and HBV-DeCi patients. Notably, the variation of Ct values in HCC patients (SD of GNB4 = 3.99, SD of Riplet = 4.74) were significantly greater than those in HBV-DeCi patients (SD of GNB4 = 1.78, SD of Riplet = 0). It is indicated that the Ct value may become an indicator of HCC.

**TABLE 2:** Statistical features of Ct values of genes GNB4, Riplet and the endogenous gene β-actin.

		GNB4		Riplet		β-actin	
		NC	HCC	NC	HCC	NC	HCC
mean		44.46	37.44	45.00	38.49	30.53	27.80
95% Confidence Interval	upper	45.52	39.29	NA	40.68	30.79	28.68
	lower	43.41	35.60	NA	36.30	30.28	26.92
5% trimming mean		45.00	37.36	45.00	38.16	30.52	27.94
Median		45.00	37.74	45.00	38.23	30.50	27.66
Standard deviation		1.78	3.99	0.00	4.74	0.55	1.89
Minimum		39.10	30.32	45.00	30.05	29.40	24.03
Maximum		45.00	45.00	45.00	45.00	31.18	31.29
Range		5.90	14.68	0.00	14.95	1.78	7.26
Quartile distance		0.00	3.80	0.00	10.38	0.51	2.79
Skewness		−3.32	0.09	45.00	0.16	−1.03	−0.03
Kurtosis		11.00	−0.12	45.00	−0.94	0.64	−0.39

**FIGURE 4:**
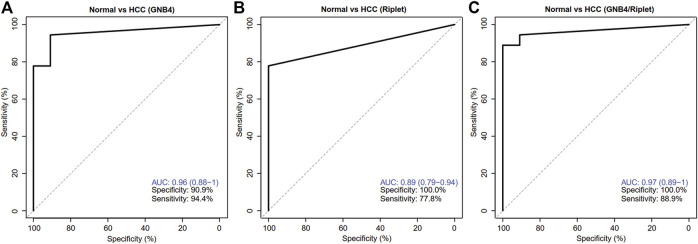
Clinical performance of GNB4 and Riplet combined to detect HCC. **(A)**, GNB4 was alone to detect HCC. **(B)**, Riplet was alone to detect HCC. **(C)**, GNB4 and Riplet were combined to detect HCC. Normal means control group patients (HBV-DeCi patients).

ROC analysis was carried out on the HCC and HBV-DeCi patients. The results were shown in Figure 4: When GNB4 was detected alone, the specificity was low (90.9%), and when Riplet was detected alone, the sensitivity was low (77.8%). The combined detection of the two genes has a maximum AUC value of 0.97 (0.89–1) for distinguishing between HCC and HBV-DeCi patients plasma, with a sensitivity of 88.9% and a specificity of 100% at the maximum AUC. It can be suggested that the combined detection of GNB4 and Riplet gene methylation levels can distinguish HCC from HBV-DeCi patients plasma.

### CTC combined with methylation detection of GNB4 and Riplet can improve the efficacy of early diagnosis of HCC

**FIGURE 5:**
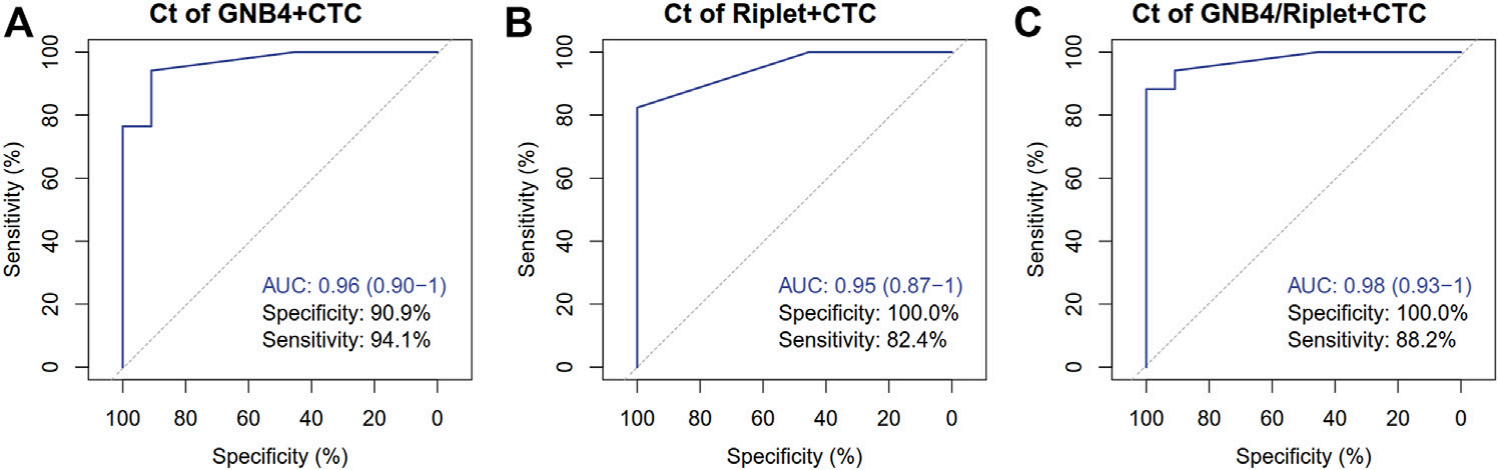
Clinical performance of CTC Combined with GNB4 and Riplet gene to detect HCC. **(A)**, GNB4 and CTC were combined to detect HCC. **(B)**, Riplet and CTC were combined to detect HCC. **(C)**, GNB4/Riplet and CTC were combined to detect HCC.

In order to verify the clinical diagnostic efficacy of CTC combined with GNB4 and Riplet gene methylation detection in plasma for HCC patients, ROC analysis was further performed for CTC count detection combined with GNB4 and Riplet gene methylation detection in the above two groups of patients, and the results were shown in Figure 5: The specificity of CTC combined with GNB4 was 90.9%, and that of CTC combined with Riplet was 100%. When CTC combined with two genes, the AUC value was significantly increased to 0.98 (0.93–1), the sensitivity was 88.2%, and the specificity was 100%. These results suggest that CTC combined with methylation detection of GNB4 and Riplet can improve the efficacy of early diagnosis of HCC.

## Discussion

The analysis of blood for CTCs or cfDNA called “liquid biopsy” has opened new avenues for cancer diagnostics, including early detection of tumors, improved risk assessment and staging, as well as early detection of relapse and monitoring of tumor evolution in the context of cancer therapies (Alix-Panabières et al., 2021; Ignatiadis et al., 2021). Also, the application of genomic profiling assays using plasma circulating tumor DNA (ctDNA) is rapidly evolving in the management of patients with advanced solid tumors (Cheng et al., 2021). However, currently, research on CTC combined with cfDNA in the early diagnosis of HCC has not been found. In our study, We developed a novel test that CTC count combined with GNB4/Riplet methylation detection and showed its high performance for early diagnosis of HCC.

Although CTC counts are rare in blood, several methods have been developed to find and show global features of tumor characteristics in HCC patients. Furthermore, it can provide a low risk means of diagnosis and guiding treatment (Ogle et al., 2016). In our study, we have found that a small number of CTCs can also be detected in HBV-DeCi patients, but the number is significantly lower than that in HCC patients. While there was no significant difference in CTC count for HCC patients with different degrees of differentiation. Quantitative abundance of CTCs, as well as biological characteristics and genomic heterogeneity among the CTCs, can predict disease prognosis and response to therapy in patients with HCC (Ahn et al., 2021). We also have found that the sensitivity and specificity of CTC for HCC detection were 70.6% and 90.9% (AUC = 0.81), it showed that CTC diagnostic test can have the good clinical application efficacy.

Early cancer detection by cfDNA faces multiple challenges: low fraction of tumor cfDNA, molecular heterogeneity of cancer, and sample sizes that are not sufficient to reflect diverse patient populations (Johnson et al., 2022). The novel method of cfMethyl-Seq can approach to address these challenges, for cost-effective sequencing of the cfDNA methylome (with>12-fold enrichment over whole genome bisulfite sequencing in CpG islands), and a computational method to extract methylation information and diagnose patients (Stackpole et al., 2022). A large clinical cohort also have demonstrated that the utility of ctDNA methylation markers in the diagnosis, surveillance, and prognosis of HCC (Xu et al., 2017). In our study, with the MSP method, we have found that the combined detection of GNB4 and Riplet gene methylation levels can distinguish HCC from HBV-DeCi patients plasma. GNB4 gene encodes the β subunit of the heterotrimer guanine nucleotide-binding proteins (G proteins), which plays a crucial role in signal transduction. It has been reported that GNB4 was identified as a potential target silenced by DNA methylation *via* DNA methyltransferase 3B (DNMT3B), which can promote the growth of breast cancer cells (Wang et al., 2018). Riplet (also known as RNF135 gene), acts as the role of E3 ubiquitin ligase, which is to suppress the antitumor immune response through modulating Th1 and CTLs in T cells (Iwamoto et al., 2022). Our research have shown that the methylation mean Ct values of GNB4 gene and Riplet gene in HCC patients are respectively as 37.44 and 38.49, Besides, they are significantly different between HCC and HBV-DeCi patients.

In a systematic review of 112 studies of the accuracy of liquid biopsy analysis, it have been found that assays for CTCs and cfDNA might aid in determining patient prognoses and monitoring HCC, although there is a risk of bias in these studies (Chen et al., 2020; Temraz et al., 2022). As there are few typical clinical characteristics during the early stage of the disease, early diagnosis of HCC is very challenging. However, CTCs or ctDNA can carry tumor-specific information. Therefore, the detection and analysis of CTCs or ctDNA can provide evidence for the early diagnosis of HCC and guide treatment (Vaidyanathan et al., 2018; Li et al., 2020). In our research, we developed a novel test that CTC count combined with GNB4/Riplet methylation detection, the AUC value was significantly increased to 0.98 (0.93–1), the sensitivity was 88.2%, and the specificity was 100%. It suggested that CTC combined with methylation detection of GNB4/Riplet can improve the efficacy of early diagnosis of HCC.

## Conclusion

We demonstrated that CTC diagnostic test can have the good clinical application efficacy. Although a small number of CTCs can be detected in HBV-DeCi patients, the number is significantly lower than that in HCC patients. Besides, the GNB4 and Riplet gene methylation levels were significantly increased in HCC patients. While CTC count combined with GNB4/Riplet methylation detection, it can greatly improve the efficacy of early diagnosis of HCC. Thus, our study has developed a novel test that CTC count combined with GNB4/Riplet methylation detection and showed its high performance for early diagnosis of HCC.

## Data Availability

The original contributions presented in the study are included in the article/supplementary material, further inquiries can be directed to the corresponding authors.
